# Evaluating Randomized Assessment Timing Design in a Simulation Study

**DOI:** 10.1093/geroni/igaf122.3580

**Published:** 2025-12-31

**Authors:** Wenshan Yu, Valerie Smith, Sarah Peskoe

**Affiliations:** Duke University, Durham, North Carolina, United States; Duke University, Durham, North Carolina, United States; Duke University, Durham, North Carolina, United States

## Abstract

Longitudinal studies are vital in aging research because they enable researchers to trace trajectories of risk factors and outcomes, such as pain, physical and cognitive function, and biomarkers. In a typical longitudinal design, follow-up time is predetermined and fixed for every individual to ensure that differences in outcomes can be attributed to true temporal changes rather than variability in measurement timing, and to prevent time-related factors from being conflated with treatment or group effects. While this approach maintains internal validity, it can also limit the ability to explore alternative follow-up schedules that might yield richer information or greater efficiency. We propose a randomized follow-up design, in which the timing of some follow-up measurements is randomized across subjects within a longitudinal study. This approach has the potential to better estimate the underlying trajectory of change. Through a series of simulation studies, we demonstrate that the proposed design can substantially reduce bias when estimating between-group differences and when extrapolating to time points not covered in a fixed-time design. It also improves the overall mean squared error in predicting outcomes across all time points, when higher-order terms in the trajectory cannot be estimated due to insufficient data. This design may be valuable for both clinical trials and prospective observational studies in settings where the trajectory pattern is unknown at the design stage. The promising simulation results motivate further application to real data, to assess the utility of the design in reducing participant burden in data collection while increasing statistical power for trajectory estimation.

